# Supplementary material for: Identification of photoperiod-induced specific miRNAs in the adrenal glands of Sunite sheep (Ovis aries)
Source: Front Vet Sci. 2022 Jul 22;9:888207. doi: 10.3389/fvets.2022.888207 (PMC9354845; doi:10.3389/fvets.2022.888207)
Supplement: Supplementary file 1 [file Data_Sheet_1.ZIP › Supplementary file/miRDeep2 file.zip!/miRDeep2 file/Know_Structure/SPLP42A2/oar-mir-99a.pdf]

miRBase precursor	: oar-mir-99a
Total read count	: 765195
oar-miR-99a read count	: 764619
remaining reads	: 576

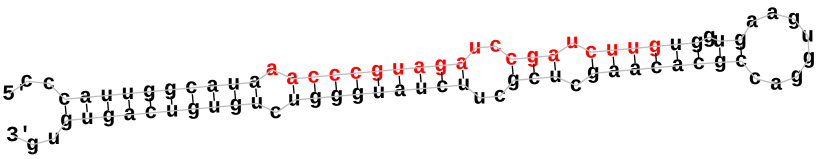

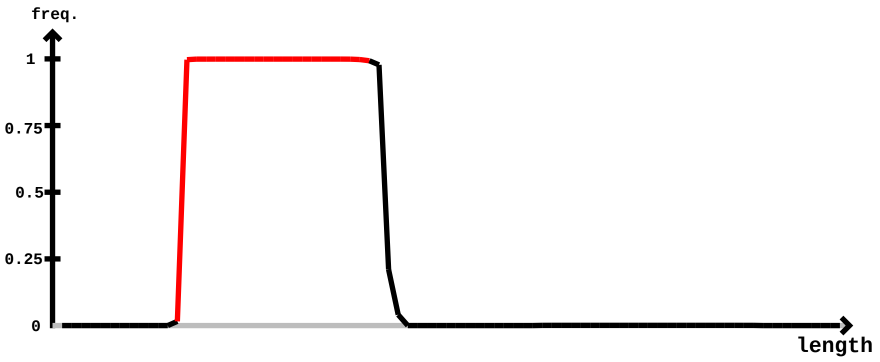

oar-miR-99a

5'- cccauuggcauaaacccguagauccgaucuuguggugaaguggaccgcacaagcucgcuucuaugggucugugucagugug -3' exp

..((((((((((.(((((((((..(((.((((((.((........)))))))).)))..))))))))).))))))))))..	reads	mm	sample
..........uaaacccguagauccgaucuugu..	2	0	seq
..........uaaacccguagauccgaucuugug...	1	0	seq
...........aaacccguagauccgau...	1	0	seq
...........aaacccguagauccgauc..	2	0	seq
...........aaacccguagauccgaucu...	21	0	seq
...........aaacccguagauccgaAcu...	1	1	seq
...........aaacccguagauccgaucuu..	59	0	seq
...........aaacccguagauccgaucuug...	193	0	seq
...........aaacccguagauccgaucuugu..	11494	0	seq
...........aaacccguagauccgaAcuugu..	3	1	seq
...........aaacccguagauccgaucuugug...	243	0	seq
...........aaacccguagauccgaAcuugug...	2	1	seq
...........aaacccguagauccgaucuugugg..	30	0	seq
............aacccguagauccgauc..	190	0	seq
............aacccguagauccgaAc..	1	1	seq
............aacccguagauccgaAcu...	88	1	seq
............aacccguagauccgaucu...	1061	0	seq
............aacccguagauccgaAcuu..	165	1	seq
............aacccguagauccgaucuu..	3235	0	seq
............aacccguagauccgaAcuug...	1041	1	seq
............aacccguagauccgaucuug...	10452	0	seq
............aacccguagauccgaAcuugu..	15210	1	seq
............aacccguagauccgaucuugu..	559753	0	seq
............aacccguagauccgaucuugug...	102002	0	seq
............aacccguagauccgaAcuugug...	26615	1	seq
............aacccguagauccgaucuugugg..	30321	0	seq
............aacccguagauccgaAcuugugg..	677	1	seq
............aacccguagauccgaucuuguggu...	28	0	seq
............aacccguagauccgaAcuuguggu...	2	1	seq
.............acccguagauccgaucuu..	3	0	seq
.............acccguagauccgaAcuug...	10	1	seq
.............acccguagauccgaucuug...	11	0	seq
.............acccguagauccgaucuugu..	653	0	seq
.............acccguagauccgaAcuugu..	173	1	seq
.............acccguagauccgaAcuugug...	535	1	seq

oar-miR-99a

cccauuggcauaaacccguagauccgaucuuguggugaaguggaccgcacaagcucgcuucuaugggucugugucagugug
.............acccguagauccgaucuugug... 155 0 seq
.............acccguagauccgaAcuugugg.. 38 1 seq
.............acccguagauccgaucuugugg.. 39 0 seq
..............cccguagauccgaucuu.. 1 0 seq
..............cccguagauccgaucuugu.. 24 0 seq
..............cccguagauccgaAcuugug... 1 1 seq
..............cccguagauccgaucuugugg.. 2 0 seq
...............ccguagauccgaucuugu.. 29 0 seq
...............ccguagauccgaAcuugu.. 2 1 seq
...............ccguagauccgaAcuugug... 1 1 seq
...............ccguagauccgaucuugug... 7 0 seq
...............ccguagauccgaucuugugg.. 4 0 seq
................cguagauccgaucuugu.. 26 0 seq
................cguagauccgaucuugug... 9 0 seq
................cguagauccgaucuugugg.. 3 0 seq
....caagcucgcuucuauggguc............ 1 0 seq
....caagcucgcuucuaugggucu........... 3 0 seq
....caagcucgcuucuaugggucug.......... 21 0 seq
....caagcucgcuucuaugggucugu......... 525 0 seq
....caagcucgcuucuaugggucugug........ 5 0 seq
....aagcucgcuucuaugggucugu......... 21 0 seq
